# High-Intensity Ultrasound Treatment on Soy Protein after Selectively Proteolyzing Glycinin Component: Physical, Structural, and Aggregation Properties

**DOI:** 10.3390/foods9060839

**Published:** 2020-06-26

**Authors:** Wenjie Xia, Siyi Pan, Zhe Cheng, Yan Tian, Xingjian Huang

**Affiliations:** 1 College of Food Science and Technology, Huazhong Agricultural University, Wuhan 430070, China; wenjie.xia@wur.nl (W.X.); pansiyi@mail.hzau.edu.cn (S.P.); zhechengfood@163.com (Z.C.); tianyan@webmail.hzau.edu.cn (Y.T.); 2 Key Laboratory of Environment Correlative Dietology, Ministry of Education, Wuhan 430070, China; 3 Physics and Physical Chemistry of Foods, Wageningen University & Research, Bornse Weilanden 9, 6708WG Wageningen, The Netherlands

**Keywords:** high-intensity ultrasound, selective proteolysis, pepsin, soluble aggregates, gelation

## Abstract

In this study, a novel method called selective proteolysis was applied to the glycinin component of soy protein isolate (SPI), and a degraded glycinin hydrolysate (DGH) was obtained. The effects of high-intensity ultrasound (HIU) treatment (20 kHz at 400 W, 0, 5, 20, and 40 min) on the physical, structural, and aggregation properties of DGH were investigated with the aim to reveal the influence of the selectively hydrolyzing glycinin component on the HIU treatment of soy protein. The effects of HIU on DGH and a control SPI (CSPI) were both time-dependent. HIU induced the formation of soluble aggregates in both samples at an early stage, while it dissociated these newly formed aggregates after a longer duration. Selectively hydrolyzing glycinin contributed to the soluble aggregation by exposing the compact protein structure and producing small protein fractions. The larger extent of hydrophobic interactions and disulfide bonds imparted a higher stability to the soluble protein aggregates formed in DGH. As a result, DGH displayed more ordered secondary structures, a higher solubility, and better gelling properties after the HIU treatment, especially at 20 min. The results of this study will be beneficial to the scientific community as well as industrial application.

## 1. Introduction

High-intensity ultrasound (HIU), which has frequency in the range of 20–100 kHz and power in the range of 10–1000 W/cm^2^, has drawn considerable attention recently as a promising nonthermal processing method with little impact on the environment [1]. Due to its cavitation, shear stress, dynamic agitation, and turbulence, as well as its promotion of some chemical reactions, HIU has the ability to alter the properties of biopolymers physically or chemically [2]. The ultrasonic treatment of soy protein has been studied in the last decade, since soy protein is one of the most promising plant proteins in the food industry. For example, Jambrak et al. [3] found that commercial soy protein isolate (SPI) and soy protein concentrate (SPC) showed a smaller particle size but higher solubility and apparent viscosity after sonication. Arzeni et al. [4] found that HIU could reduce the viscosity and particle size of commercial SPI. Upon their treatment, the free sulfhydryl groups remained unchanged. On the other hand, Hu et al. [5] reported that the HIU treatment increased the free sulfhydryl content of commercial SPI as well as its solubility and surface hydrophobicity. Besides this, HIU could also induce the dissociation and/or aggregation of protein molecules. Zheng et al. [6] found that HIU could increase the aggregates in native SPI while dissociating the aggregates in alcohol-denatured and heat moisture-denatured SPI.

The divergent results in the literature reveal that the effects of implementing HIU treatment on soy protein depend on not only the treatment conditions but also the intrinsic properties of the protein. Most of the previous studies were conducted with total protein isolates or concentrates, with the drawback of limiting the knowledge of phenomena due to the important complexity of the protein composition. Like many food proteins, soy protein is a multicomponent biomacromolecule which consists of β-conglycinin and glycinin as the two main components. They are the vital intrinsic factors that affect the physicochemical properties, structural characteristics, and aggregation behaviors of soy protein. It has been found that, compared with isolated β-conglycinin, HIU had minor effects on isolated glycinin in pH 7.0 [7], and the effects of HIU on the isolated glycinin varied with different ionic strengths [2]. Therefore, how these soy protein components, especially glycinin, influence the HIU treatment of soy protein is worth further study.

When it comes to the enzymolysis of protein, the most common method is limited proteolysis. Through the degree of hydrolysis (DH), limited proteolysis allows researchers to obtain reproducible results and avoid excessive protein hydrolysis [8]. However, since DH only indicates the proportion of cleaved peptide bonds, when the substrate is a multicomponent protein like soy protein, limited proteolysis can hardly control and determine which specific component or subunit of protein is being hydrolyzed. As a result, a novel method called selective proteolysis has been studied in recent years [9,10,11,12]. Differently from traditional proteolysis, selective proteolysis is more precise and controllable in terms of the acting sites, by which one can selectively and exclusively degrade a target component in soy protein without affecting the others. In general, glycinin has more compact globular conformation and lower molecular flexibility than β-conglycinin, which often limits the functional properties of soy protein. Li et al. [9,10] found that selectively hydrolyzing glycinin could facilitate soy protein to form interfacial films with high viscoelastic moduli, and thus its emulsifying properties improved. Our previous study found that decreasing the β-conglycinin component by selective proteolysis was beneficial to the formation of longer fibrils in the subsequent protein fibrillation process [12].

To the best of our knowledge, few studies have investigated the effects of HIU on protein hydrolysates, let alone the hydrolysates prepared by selective hydrolysis. Against the above-mentioned background, selective proteolysis was used in this study to alter the composition of soy protein substrate and obtain a soy protein hydrolysate in which the glycinin component was selectively degraded (referred to as degraded glycinin hydrolysate, DGH). Then, the DGH was treated by HIU (20 kHz, 400 W) for different times (0, 5, 20, and 40 min), together with a control SPI sample (referred to as CSPI, blank group), which was prepared similarly except for adding protease. By comparing the changes in their physical, structural, and aggregation properties during HIU processing, this study for the first time reveals how the selectively hydrolyzing glycinin component affects the HIU treatment of soy protein, which will be beneficial to the scientific community as well as the industrial application of vegan food based on soy protein hydrolysates.

## 2. Materials and Methods

### 2.1. Materials

Cold defatted soy flour (protein concentration 56% (N × 6.25) (dry based) was obtained from Qi Tian Biotechnology Co., Ltd. (Henan, China). A Lowry assay kit was purchased from Labaide Biosciences Co. (Shanghai, China). Papain (21 units/mg), antipain (Sigma nr A-6191), and 1-anilino-8-naphthalene-sulfonate (ANS) were purchased from Sigma-Aldrich Co. (St. Louis, MO, USA). Deionized distilled water (DDW) was prepared by a Milli-Q Direct 8 water purification system (Merck Millipore Co., Burlington, NJ, USA). All the other reagents used were of analytical grade.

### 2.2. Preparation of Native Soy Protein Isolate (SPI)

According to our previous study [12], defatted soy flour was dispersed in DDW (1:20 w/w) and adjusted to pH 8.0 with 2 mol/L NaOH. The dispersion was stirred at room temperature (25 °C) for 1.5 h before centrifuging at 9000× *g* 4 °C for 20 min. The supernatant was adjusted to pH 4.5 with 2 mol/L HCl and centrifuged (9000× *g*, 4 °C) again for 20 min. The obtained SPI precipitate was re-dissolved (1:4 w/w) in DDW and adjusted to pH 7.0 before freeze drying. The protein concentration of the obtained SPI was 92.85% ± 0.35% (N × 6.25) according to the Dumas measurement.

### 2.3. Selective Proteolysis on Glycinin Component

The selective proteolysis on the glycinin component of SPI was based on the method of Li et al. [9]. The lyophilized SPI (25 g) was well dispersed in 500 mL of DDW and then adjusted to pH 2.0 and 37 °C. Pepsin was added to the SPI dispersion with an E/S ratio of 0.02%, and the enzymatic reaction was carried out by incubating at pH 2.0 and 37 °C for 40 min. After that, the reaction was terminated by neutralizing the dispersion to pH 7.0 and boiling for 5 min. The hydrolysate was then dialyzed against DDW in the molecular porous membrane tubing (MWCO: 0.5 kDa; Spectrum Medical Industries Inc., USA) at 4 °C for 48 h before freeze drying. This lyophilized sample was referred to as degraded glycinin hydrolysate (DGH). The degree of hydrolysis (DH) of the DGH was 1.87% ± 0.08% according to the pH-STAT measurement [13]. A control SPI sample (CSPI) was prepared in the same manner (incubated for 40 min) without the addition of pepsin.

### 2.4. High-Intensity Ultrasound (HIU) Treatment of Proteins

The DGH and CSPI dispersions (10%, w/v) were prepared by stirring lyophilized powder into DDW for 1 h. An ultrasound processor (Scientz Biotechnology Co. Ltd., Ningbo, China) with a 0.636 cm diameter titanium probe was used. The protein dispersions (25 mL) were sonicated (20 kHz, 400 W) for 0, 5, 20, and 40 min (4 s: 2 s on/off cycles) in 50 mL beakers, which were immersed in an ice-water bath. After sonication, all the samples were lyophilized and stored in airtight containers. The actual power and intensity of the HIU treatment in the present study were measured by the method of Arzeni et al. [4]. DDW (25 mL) was used for the acoustic power estimation. The C_p_ of water is 4.2 J/(g·K). The ultrasound intensity in this research was 34–37 W/cm^2^ (0.43–0.47 W/cm^3^).

### 2.5. Sodium Dodecyl Sulfate-Polyacrylamide Gel Electrophoresis (SDS-PAGE) Analysis

SDS-PAGE was performed based on the method of Laemmli [14], using 12% separated gel and 5% stacking gel. The sample solutions (2 mg/mL) were prepared with an SDS-PAGE sample buffer (0.0625 mol/L Tris-HCl (pH 8.0) containing 10% (v/v) glycerol, 5% (v/v) 2-mercaptoethanol, 2% (w/v) Sodium dodecyl sulfate (SDS), and 0.25% (w/v) bromophenol blue). Aliquots (15 μL) of the sample solution was loaded per well after incubating at 95 °C for 5 min. The gel was stained with Coomassie Brilliant Blue R-250 for 1 h and destained with 20% methanol and 10% acetic acid mixed solution. The gel was scanned and analyzed by a GS-900 calibrated densitometer with the Image Lab software (Bio-Rad Laboratories, Inc., Hercules, CA, USA).

### 2.6. Determination of Protein Solubility

The protein solubility was determined by the method of Huang et al. [15] with modifications. The lyophilized samples were added to the DDW (10 mg/mL) and stirred for 1 h at 25 °C. Then, the dispersions were centrifuged at 10,000 g for 20 min. The protein content in the dispersion and the protein content in the supernatant after centrifugation both were determined by the Lowry method, using bovine serum albumin as standard. The absorbance at 750 nm was measured by a Bio-spectrophotometer (Eppendorf Co. Ltd., Hamburg, Germany). The protein solubility was calculated as follows: protein solubility (%) = (protein content of the supernatant)/(total protein content before centrifugation) × 100%.

### 2.7. Dynamic Light Scattering (DLS)

The particle size of the samples were determined by dynamic light scattering (DLS) using a Nano-ZS Zetasizer (Malvern Instrument Ltd., Worcestershire, UK) [2]. The sample supernatants mentioned in Section 2.6 were diluted to 0.15 mg/mL with DDW to avoid multiple scattering and then measured in 1 cm × 1 cm disposable cuvettes (model: DTS0012) at 25 °C. The backscattering angle was 173°, the refractive index (water) was 1.333, and the equilibration time was 60 s.

### 2.8. Surface Hydrophobicity (H_0_) Measurement

Based on the method of Kato et al. [16], the lyophilized samples were dissolved in 0.01 mol/L of phosphate buffer (pH 7.0) to a series of protein concentrations (0.05, 0.1, 0.2, 0.5, 1, and 2 mg/mL). Then, each sample solution (5 mL) was mixed with 40 μL of 1-anilino-8-naphthalene sulfonate (ANS) solution (8.0 mmol/L in 0.01 mol/L phosphate buffer, pH 7.0). The fluorescence intensity (FI) was measured by a F-4600 fluorescence spectrophotometer (Hitachi, Japan) at wavelengths of 390 nm (excitation) and 470 nm (emission). The slope of the FI vs. protein concentration plot (calculated by a linear regression analysis) was used as the index of surface hydrophobicity (H_0_).

### 2.9. Circular Dichroism Spectra Measurement

The circular dichroism spectra were collected in the Far-UV range (190~250 nm) by a J-1500 circular dichroism spectropolarimeter (Jasco Corp., Tokyo, Japan) at 25 °C. The sample supernatants in Section 2.6 were diluted to 0.15 mg/mL and measured in 0.1 cm quartz CD cuvettes. The scan rate, response, and bandwidth were set as 50 nm/min, 4 s, and 1.0 nm, respectively [6]. The recorded spectra were an average of three scans and corrected by subtracting the spectrum of the protein-free buffer. The proportions of the four secondary structures (α-helix, β-sheet, β-turn, random coil) were derived from Yang’s equation. A mean value of 110 for the amino acid residue was used in the calculation.

### 2.10. Intrinsic Fluorescence Spectra Measurement

The intrinsic fluorescence spectra of the samples were obtained by a F-4600 fluorescence spectrophotometer (Hitachi, Japan) at 25 °C, based on the method of Jiang et al. [17]. The sample supernatants mentioned in Section 2.6 were diluted to 0.075 mg/mL with DDW and then excited at 290 nm. The emission spectra were recorded from 300 to 400 nm. Both the excitation (Ex) slit and emission (Em) slit were set as 5 nm, and the scan speed was 240 nm/min.

### 2.11. FT-Raman Spectra Measurement

The FT-Raman spectra were collected on lyophilized samples by an INVIA laser Raman spectrometer (Renishaw, UK) under the following settings: laser wavelength, 1064 nm; laser power, 1 W; spectral resolution, 4 cm^−1^; number of scans, 800. The obtained spectra were normalized by using a phenylalanine band at around 1004 cm^−1^ as an internal standard, since its intensity was neither sensitive to the conformational changes nor the microenvironmental changes [18].

### 2.12. Scanning Electron Microscopy (SEM)

The morphology of the lyophilized protein samples was observed by a scanning electron microscope (SU8000, Hitachi, Tokyo, Japan). Before using the scanning electron microscopy, the samples were ground by a mortar and pestle and then coated with gold.

### 2.13. Low-Amplitude Oscillatory Measurement

The thermal gelation of the sample dispersion (9% w/v, pH 7.0) was performed with a MCR302 rheometer (Anton Paar, Austria) with a sandblast concentric cylinder (CC17) and a solvent trap. The measurement was conducted within the linear viscoelastic region at a constant strain of 1% and a frequency of 1 Hz. The temperature sweep was as follows: heating from 20 °C to 95 °C at 3 °C/min, holding at 95 °C for 30 min, cooling from 95 °C to 20 °C at 3 °C/min, holding at 20 °C for 5 min. The storage modulus (G′) and loss modulus (G″) were recorded as a function of time.

### 2.14. Statistical Analysis

All the analytical determinations were performed in triplicate and the results were presented as the means ± standard deviation. The figures were plotted by the Origin 2018 software (OriginLab, Northampton, MA, USA). An ANOVA (one-way analysis of variance) and Duncan′s test at *p* < 0.05 were conducted by SPSS 25.0 (SPSS Inc., USA) to evaluate the statistical significance of the differences among the means.

## 3. Results and Discussion

### 3.1. SDS-PAGE

**Figure 1:**
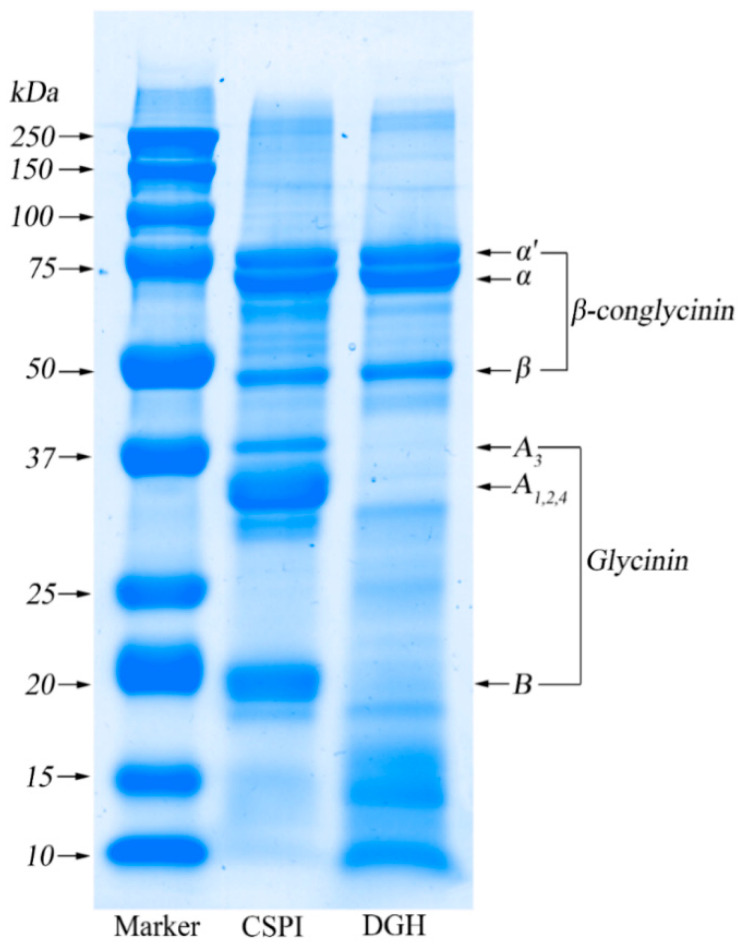
SDS-PAGE patterns of control soy protein isolate (SPI) (CSPI, blank group) and degraded glycinin hydrolysate (DGH, contrast group).

As shown in Figure 1, under the reducing condition (with 2-mercaptoethanol) the CSPI showed a typical SDS–PAGE profile of soy protein, which mainly consisted of individual subunits of β-conglycinin and glycinin. As a trimer, β-conglycinin consisted of three subunits: α’ (~80 kDa), α (~70 kDa), and β (~50 kDa). On the other hand, the glycinin component is a hexamer which contained an acidic subunit (A_1–4_) (32–40 kDa) and a basic subunit (B) (~20 kDa). It was clear that the bands for the acidic subunit and the basic subunit all disappeared in DGH, while the bands for the subunits of β-conglycinin still existed. In addition, the wide and vague bands below each subunit of glycinin appeared in DGH, which represented the small fractions with different molecular weights that derived from the degradation of glycinin. According to the densitometric analysis (Table 1), the relative content of all the glycinin subunits decreased dramatically from 60.7% in CSPI to only 2.5% in DGH, while the relative content of the small fractions increased significantly from 5.8% in CSPI to 38.5% in DGH. Correspondingly, the proportion of all the β-conglycinin subunits increased from 33.5% in CSPI to 59.0% in DGH. Achieving this selectivity relies on the enzymatic reaction conditions as well as the enzyme. On the one hand, glycinin is known to be denatured preferentially in an acidic pH [19]. Therefore, compared with β-conglycinin, the glycinin component would be denatured to a much higher extent in the present reaction conditions (pH 2.0, 37 °C) and time (40 min), which made the cleavage sites in glycinin more accessible to the enzyme. On the other hand, pepsin was chosen because it shows selectivity to glycinin and it is optimally active at pH 2.0 [11]. Owing to these two aspects, the glycinin component of SPI was hydrolyzed successfully in the present study, while the β-conglycinin component was not affected.

### 3.2. Solubility

**Figure 2:**
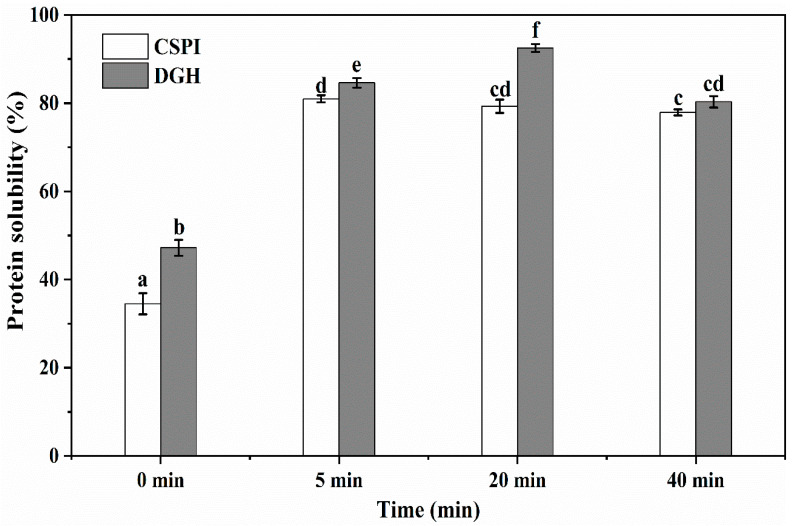
Effect of high-intensity ultrasound treatment (20 kHz at 400 W for 0, 5, 20, 40 min) on the protein solubility of CSPI and DGH. Different lowercase letters (a, b, c…) indicate significant differences between samples, at *p* < 0.05 using Duncan’s test. Error bars represent the standard deviations.

As shown in Figure 2, the solubility of CSPI before the HIU treatment was 34.5%, which agreed with Huang et al. [15] that the solubility of the commercial SPI after acid treatment (pH 3.0, 1 h) was about 30% at pH 7.0. The higher solubility (47.2%) showed by DGH suggested that selective hydrolysis on glycinin could increase the solubility of soy protein by breaking the peptide bonds and reducing the particle size (Section 3.3). HIU increased the solubility of DGH and CSPI significantly (*p* < 0.05) to 84.6% and 81.0% after 5 min. This improvement could be explained by two reasons: (1) the HIU treatment could reduce the particle size of proteins, which improved the protein dispersibility in the solvent and the protein–solvent interactions, so the solubility of the protein increased [4]; (2) HIU could induce the transformation of insoluble aggregates or precipitates to soluble protein aggregates, which made more protein in the soluble state [20]. Interestingly, when the HIU treatment was continued to 20 min, the solubility of the CSPI became lower, while the solubility of DGH kept increasing. The solubility of DGH reached 92.5% at 20 min, which was significantly higher (*p* < 0.05) than that of CSPI (79.3%). Since the surface-active and gelation properties require proteins to be soluble in the relevant medium, selectively hydrolyzing glycinin may facilitate the ultrasonic modification of soy protein in terms of these techno-functionalities. When the HIU treatment was prolonged to 40 min, the solubility of both the samples decreased. Similar results were found by Huang et al. [15] and Jiang et al. [17] that the solubility of commercial SPI and black bean protein could be unchanged or decreased with a further increase in ultrasonic time.

### 3.3. Particle Size

**Figure 3:**
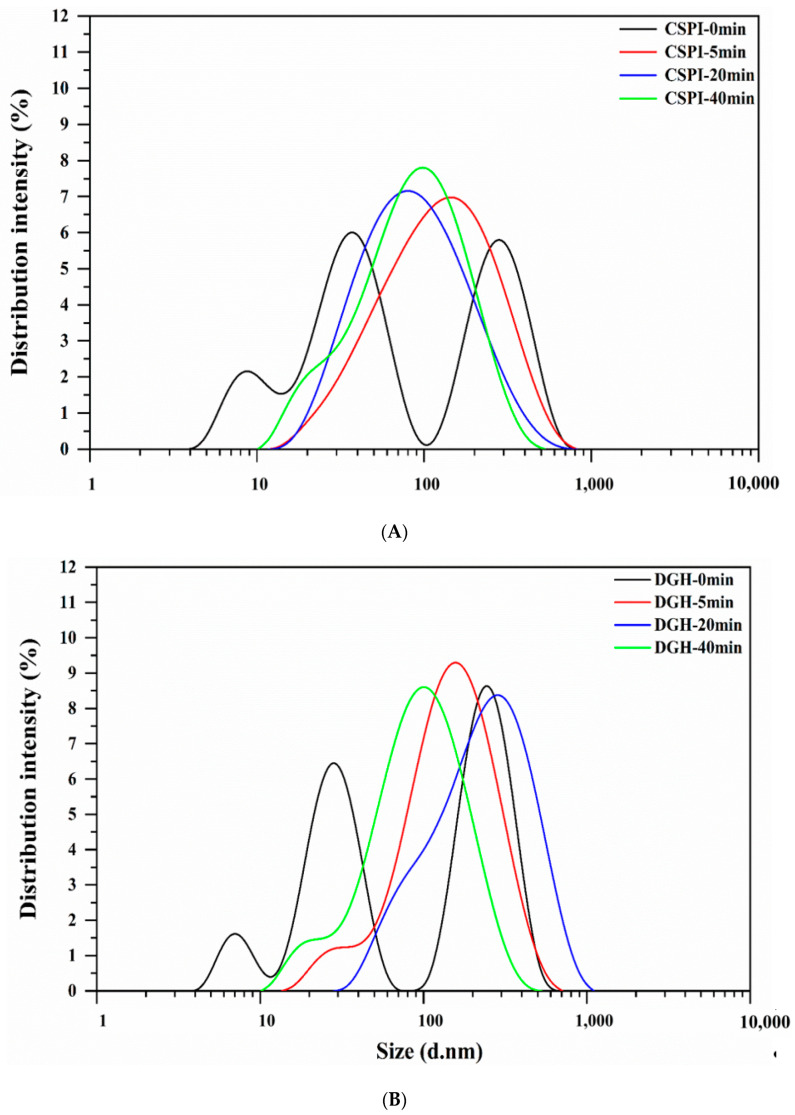
Effect of high-intensity ultrasound treatment (20 kHz at 400 W for 0, 5, 20, 40 min) on the particle size distribution of CSPI (**A**) and DGH (**B**).

As shown in Figure 3, before HIU treatment the DGH and CSPI both demonstrated a multimodal particle size distribution (PSD) with three peaks. The hydrodynamic diameter of native SPI has been reported to be around 34 nm (at pH 7.0 and 25 °C) [21], which explains the middle peak at 28–38 nm. The peak located below 10 nm could be attributed to the small protein fractions that were dissociated from SPI, while the peak at above 100 nm indicates the existence of large aggregates. Enzymatic hydrolysis shifted all three peaks of DGH to smaller sizes compared to those of CSPI, and DGH showed a lower average size and higher polydispersity index (PDI) (Table 2). After the HIU treatment, the PSD of DGH and CSPI both changed into a unimodal distribution and their PDI both declined, showing the homogenization effects of high-intensity ultrasound. Interestingly, the single peaks of the HIU-treated samples were all located at higher sizes than the peak of the native SPI (the middle one in the multimodal distribution), and their average sizes all increased. This means most of the protein that in the soluble state after HIU treatment, were existed in the form of aggregates. Combining the markedly increased solubility (Figure 2), the formation of soluble aggregates after the HIU treatment was mainly responsible for the improved solubility in the present study. Tang et al. [20] reported that the high-frequency oscillation caused by HIU (200 W, 15 kHz) could slow down the association of small unstable aggregates in soy protein dispersion while promoting the interactions between unstable aggregates and other soluble protein components (e.g., the α and α’ subunits of β-conglycinin), eventually forming soluble aggregates with a relatively stable structure [20].

When the HIU treatment was prolonged (>5 min), the average size of CSPI showed a decreasing trend. In contrast, the PSD of DGH further moved to larger sizes, with the peak centered at 295.0 nm at 20 min (Figure 3), and its average size decreased only after 40 min (Table 2). These trends displayed here were consistent with the trends showed in the solubility (Section 3.2). These phenomena reflected that HIU could induce the formation of soluble aggregates in both samples within a certain time. However, the longer duration of HIU processing would not promote further aggregation but dissociate those newly formed aggregates. Zheng et al. [6] observed that HIU (80 W/cm^2^) increased the particle size of native SPI aggregates after 10 min while reducing the particle size after 25 min. Similarly, Shanmugam et al. [22] found the soluble whey/casein aggregates and micellar aggregates were formed during the first 30 min of sonication (0.31 and 0.63 W/cm^3^), but prolonged sonication caused the partial disruption of whey proteins from these aggregates due to the continuous shear. Noticeably, the soluble aggregates in DGH continued aggregating in a relatively longer duration, and DGH nanoparticles existed as bigger aggregates with relatively uniform sizes (Table 2). It has been reported that glycinin tended to form insoluble aggregates, while β-conglycinin tended to form soluble aggregates, but the subunits from glycinin could interact with β-conglycinin to form soluble aggregates as well [23,24,25]. As shown in the SDS-PAGE (Figure 1), selective proteolysis in this work retained the β-conglycinin while disintegrating glycinin into small fractions, which may provide DGH with more “building blocks” for the soluble aggregation in the subsequent HIU treatment.

### 3.4. Surface Hydrophobicity

**Figure 4:**
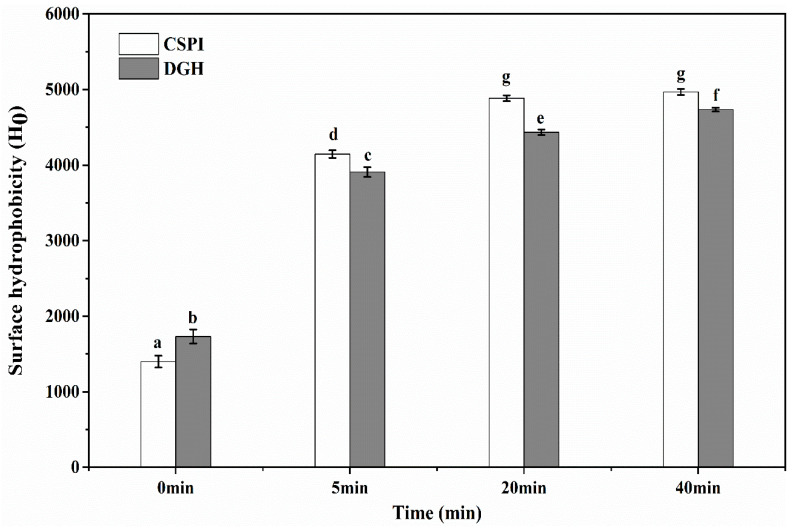
Effect of high-intensity ultrasound treatment (20 kHz at 400 W for 0, 5, 20, 40 min) on the surface hydrophobicity of CSPI and DGH. Different lowercase letters (a, b, c…) indicate significant differences between samples at *p* < 0.05 using Duncan’s test. Error bars represent the standard deviations.

As shown in Figure 4, the surface hydrophobicity (H_0_) of DGH was higher than that of CSPI before the HIU treatment. This was expected, since the enzymatic cleavage destroyed the compact structure of glycinin, which made more hydrophobic groups accessible to external fluorescence probes—i.e., ANS—thus showing a higher H_0_. HIU increased the H_0_ of DGH and CSPI significantly (*p* < 0.05), although the increasing rate tended to slow down with treatment time. By the cavitation effect of HIU, a great extent of molecular unfolding and structural changes could happen in protein molecules, which enhanced their surface hydrophobicity and promoted intermolecular aggregation [26]. Interestingly, although the H_0_ of both the samples increased, the H_0_ of DGH became lower than that of CSPI after the HIU treatment. The hydrophobic interactions have been viewed as the driving force for protein aggregation [27]; the lower H_0_ suggested that, in the simultaneous effects of HIU-induced denaturation and aggregation, more exposed hydrophobic groups in DGH participated in the hydrophobic interactions during the formation of soluble aggregates. As reported previously [28,29], partially unfolded proteins with an initially higher surface hydrophobicity could cause more extensive bonding and aggregation, which in turn entrapped the hydrophobic regions and manifested in a lower H_0_ in DGH. Furthermore, with more intermolecular hydrophobic interactions being involved, soluble aggregates in DGH should have a higher stability, which could help them to endure a relatively longer time of ultrasonic vibration and grow into a bigger size, which agreed with the observation in Section 3.3.

### 3.5. Circular Dichroism Spectroscopy

Table 3 shows the secondary structure composition of DGH and CSPI that was deduced from circular dichroism spectroscopy. Prior to the HIU treatment, DGH showed less ordered structure (α-helix and β-sheet) and more unordered structure (β-turn and random coil) than CSPI. HIU induced a significant increase in the α-helix structure, a slight increase in the β-turn structure, and a decrease in the random coil structure (*p* < 0.05). For the β-sheet structure, the opposite changes were observed: the β-sheet structure of DGH increased while that of CSPI decreased. As a result, the composition of the ordered structure in DGH turned out to be higher than that of CSPI, especially at 20 min. The highly repetitive feature of β-sheet and higher content of ordered structure could be closely correlated with the higher stability of DGH soluble aggregates. Zheng et al. [6] and Hu et al. [5] both observed an increased α-helix content and decreased β-sheet content in the HIU treatment of native SPI (80 W/cm^2^) and commercial SPI (105–138 W/cm^2^), which was consistent with the present results of CSPI. However, when the isolated β-conglycinin and glycinin were solely subjected to an equivalent ultrasonication (105–110 W/cm^2^), neither of them showed significant changes in their secondary structures [7]. These results demonstrated that when glycinin existed as a component in soy protein, its influence on the ultrasonic modification of whole protein may not be deduced by simply imposing the same treatment on the isolated glycinin.

### 3.6. Intrinsic Fluorescence Spectroscopy

**Figure 5:**
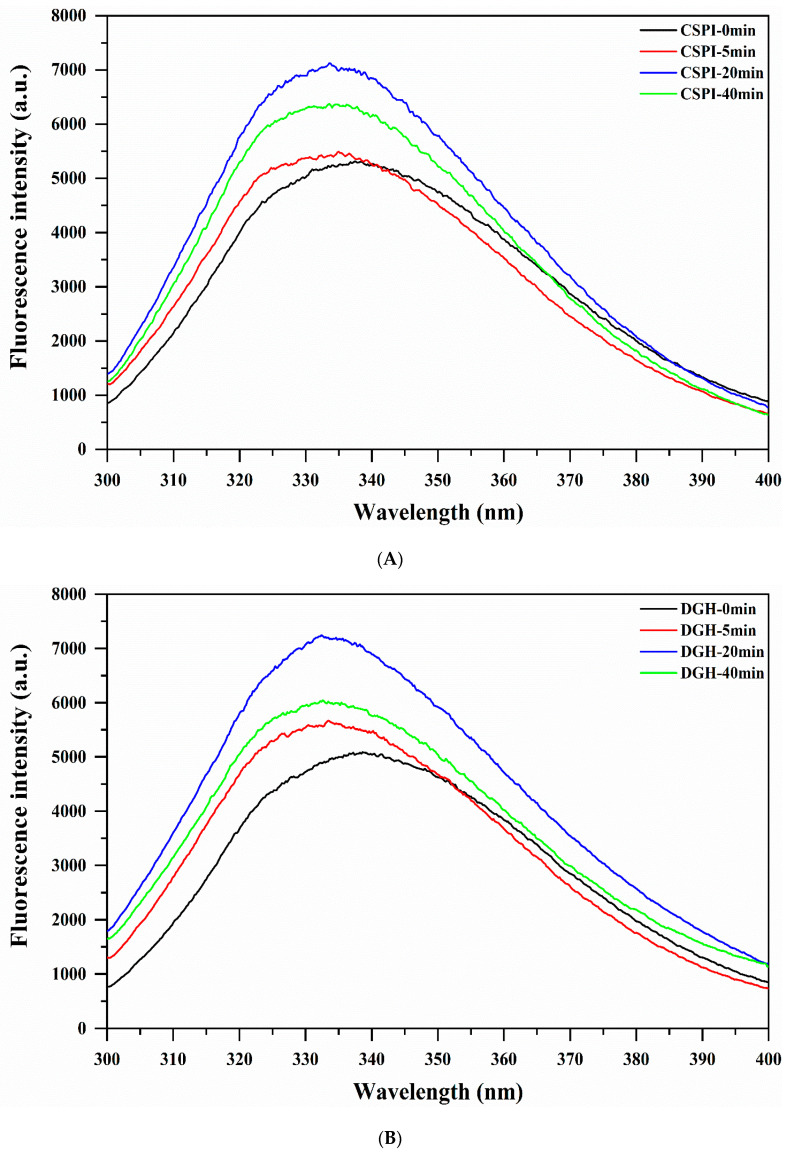
Effect of high-intensity ultrasound treatment (20 kHz at 400 W for 0, 5, 20, 40 min) on the intrinsic fluorescence emission spectra of CSPI (**A**) and DGH (**B**).

The intrinsic fluorescence spectra of protein are mainly attributed to Tryptophan (Trp) residues, which can be used for analyzing the tertiary structure. As shown in the Figure 5, the intrinsic fluorescence spectra of DGH and CSPI showed a significant blue-shift (decrease in λ_max_) and increased FI after the HIU treatment. The blue-shift of λ_max_ indicated that the microenvironment of Trp residues became less polar. Protein molecular aggregation prevented the interaction of chromophores with the quenching agent present in the solvent and hence a higher FI [30]. DGH displayed a lower λ_max_ and higher FI (except at 40 min) than CSPI after the HIU treatment (Table 4), which was consistent with the previous analysis that showed the soluble aggregation in DGH involved more hydrophobic interactions, which provided the Trp residues a more hydrophobic microenvironment. At a later stage of the HIU treatment (> 20 min), there were no significant changes in the λ_max_ of DGH and CSPI, whereas the FI of them both significantly declined (*p* < 0.05). Similar results were observed by Zhu et al. [31], who showed that the FI of walnut protein decreased with increasing sonication time while the λ_max_ was unchanged, which was an indicative of changes in the protein structure and/or aggregation state.

### 3.7. FT-Raman Spectroscopy

**Figure 6:**
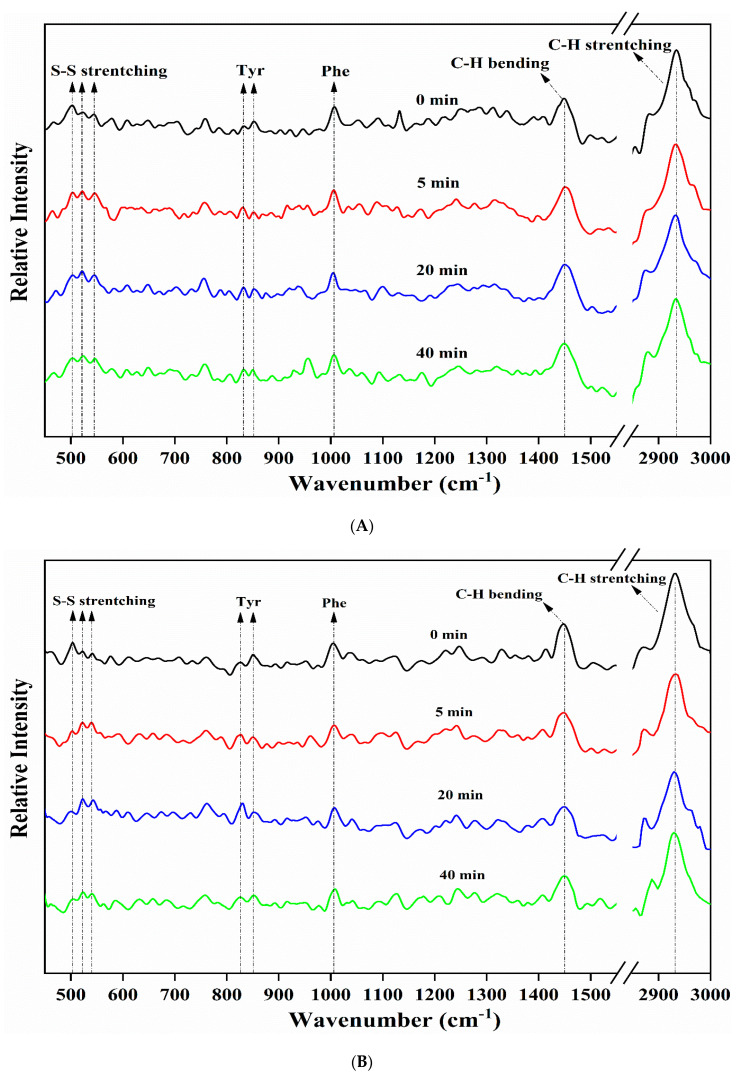
Effect of high-intensity ultrasound treatment (20 kHz at 400 W for 0, 5, 20, 40 min) on the FT-Raman spectra of CSPI (**A**) and DGH (**B**).

Disulfide exchange reactions and the formation of disulfide bonds (S-S) play crucial roles in the unfolding and functional aggregation of proteins [32]. The characteristic frequencies of S-S stretching vibrations in the Raman spectra are assigned to gauche-gauche-gauche (g-g-g, 500–510 cm^−1^), gauche-gauche-trans (g-g-t, 515–530 cm^−1^), and trans-gauche-trans (t-g-t, 535–545 cm^−1^), which are three conformation forms. As shown in Figure 6 and Table 5, the Raman peak at 500–510 cm^−1^ exhibited the highest intensity before the HIU treatment. This is consistent with a previous study, which showed that the S-S stretching vibrations of soy protein tended to be dominated by the g-g-g conformation, since it had the lowest potential energy [18]. However, the intensity of the g-g-g conformation decreased after the HIU treatment, while that of g-g-t and t-g-t conformations both increased (*p* < 0.05). It has been reported that the g-g-g conformation of disulfide bonds was negatively correlated with the surface hydrophobicity of protein, while the g-g-t conformation promoted the surface hydrophobicity [33], which was in conformity with the increased H_0_ after the HIU treatment (Section 3.4). For DGH, the intensity of the g-g-t and t-g-t conformations increased gradually in the first 20 min and then decreased after 40 min. For CSPI, significant changes (*p* < 0.05) in the S-S stretching vibrations were only observed in the first 5 min. These phenomena suggested that HIU had a more pronounced effect on the S-S stretching vibration in DGH. Each pair of acidic subunit and basic subunit in glycinin is associated with disulphide bridges [20]; selectively hydrolyzing glycinin could break these disulphide bonds and release sulfhydryl groups. Under the following ultrasonic cavitation effects, the highly reactive free radicals generated from water molecules could oxidize susceptible sulfhydryl groups to form intermolecular disulphide bonds, which might play an important role in the soluble aggregation of DGH [20,34,35]. Similarly, Lee et al. [32] found that pH shifting could cause the cleavage of disulphide bonds between glycinin subunits, which led to increased S-S bonds and higher protein solubility in the subsequent ultrasonication.

The intensity ratio of 850 to 830 cm^−1^ (I_850/830_) and the intensities of the Raman peaks near 1450 and 2935 cm^−1^ were used to reflect the microenvironment of Tyr residues and aliphatic amino acid residues, respectively [18,36]. HIU first reduced the I_850/830_, I_1450_, and I_2935_ in a short period, indicating that the microenvironment of these residues become less polar due to the protein intermolecular interactions [37]. However, as the HIU treatment was prolonged, the value of these indexes increased again, which indicated that some of the HIU-induced aggregates were disassociated under prolonged ultrasonic shear forces and turbulence, and the amino acid residues that were buried by them became exposed again. However, due to the higher stability of the aggregates, this dissociation phenomenon was obviously delayed in DGH, as these indexes only increased after 40 min.

### 3.8. Scanning Electron Microscopy (SEM)

**Figure 7:**
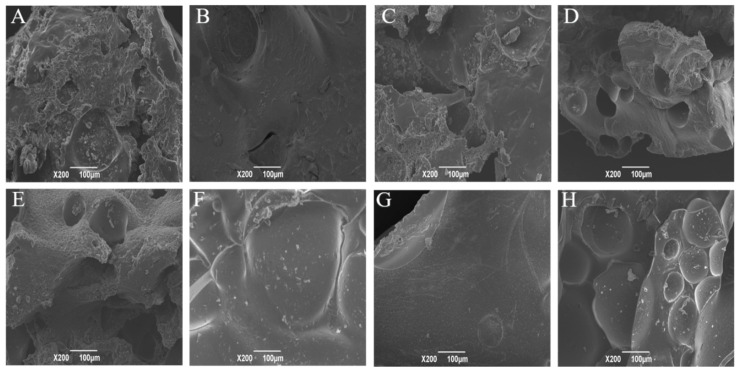
Effect of high-intensity ultrasound treatment (20 kHz at 400 W for 0, 5, 20, 40 min) on the SEM micrographs of lyophilized CSPI and DGH. (**A**) CSPI-0 min, (**B**) CSPI-5 min, (**C**) CSPI-20 min, (**D**) CSPI-40 min; (**E**) DGH-0 min, (**F**) DGH-5 min, (**G**) DGH-20 min, (**H**) DGH-40 min.

The changes in the microstructure of the lyophilized DGH and CSPI after the HIU treatment was observed by SEM. Figure 7 shows that all the samples presented in the form of massive chunks after freeze drying, but with different shapes and surface morphologies. As previously reported, larger protein aggregates with a layer block structure were observed in the sonicated legume proteins in their lyophilized state [5,38]. Before the HIU treatment, DGH displayed a complicated structure with a rough surface, which had many irregular humps and cavities of different sizes. On the other hand, CSPI showed a relatively flat structure. This provided tangible evidence of the degradation effects of selective proteolysis on the structural integrity of soy protein. The surface morphology of both samples became more flat and compact in the early stage of the HIU treatment. Interestingly, compared to CSPI, DGH showed a crystalline microstructure with a smoother surface, only having some debris, especially at 20 min. This denser microstructure of DGH should be ascribed to the smaller inhomogeneities and a larger extent of intermolecular interactions and soluble aggregation in the protein dispersion before freeze drying.

### 3.9. Gelling Property

**Figure 8:**
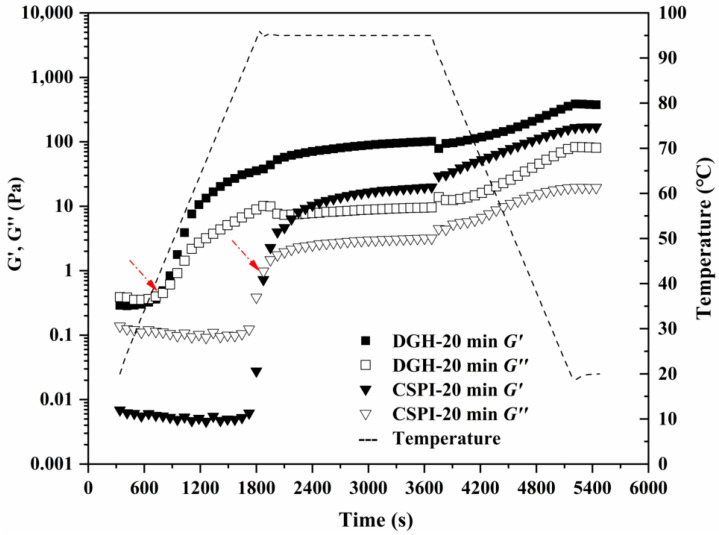
The thermal gelation profiles of DGH (rectangle) and CSPI (triangle) after being treated by HIU for 20 min. The solid symbols indicate G′, while the open symbols indicate G″. The red arrow indicates the onset gelling point.

The formation of soluble aggregates is beneficial to the gelling properties of soy proteins, since they are known as the intermediate product during the heat-induced gelation of soy proteins [20]. Therefore, the thermal dynamic gelation of DGH and CSPI after 20 min of HIU treatment were measured by the small amplitude oscillatory test. As shown in Figure 8, both samples showed a typical heat-induced gelation profile, as G′ and G″ increased after a cycle of heating and cooling (Figure 7). The cross point of G′ and G″ is usually viewed as the onset of gelation. For CSPI, the onset time was around 1900 s when the temperature reached 95 °C. The increased G′ at this temperature was related to the denaturation of glycinin component, by which more unfolded proteins interacted with each other, leading to irreversible protein aggregation and network formation [39]. However, the onset time and temperature for DGH moved much earlier to 760 s and 42 °C, respectively. This agreed with above results that the glycinin component in DGH had already been denatured after the selective hydrolysis and ultrasonication. With the existence of soluble aggregates, the initial protein network was formed earlier in the HIU-treated DGH, suggesting its improved gelling ability. Furthermore, the formed gel of the HIU-treated DGH after the temperature sweep showed a significantly higher G’ (~386 Pa) than its CSPI counterpart (~169 Pa), indicating a stiffer gel structure. This could be due to the higher solubility of DGH after the HIU treatment, which allowed more protein molecules to be incorporated into the gel network.

## 4. Conclusions

In this work, selective proteolysis specifically hydrolyzed the glycinin component in SPI, which destroyed the structural integrity and released small protein fractions. As a result, the resultant hydrolysate (DGH) showed different physical, structural, and aggregation properties under the subsequent HIU treatment (20 kHz at 400 W for 0, 5, 20, and 40 min), compared with the control SPI (CSPI). HIU induced the formation of soy protein soluble aggregates in the early stage (≤20 min), while it disassociated them after a longer duration (40 min). Compared with CSPI, the DGH nanoparticles formed soluble aggregates with a larger size and higher stability, which could be attributed to the existence of small protein fractions and the larger extent of hydrophobic interactions as well as disulfide bonds. Correspondingly, a higher solubility, more ordered secondary structures, and less polar microenvironment of amino acid residues were observed in DGH, especially at the intermediate time (20 min). After freeze drying, the HIU-treated DGH showed a denser crystalline structure with a smooth surface. The superior solubility and gelling ability showed by the HIU-treated DGH indicated the combination of selective proteolysis, and HIU could be a novel method for soy protein modification. The HIU-treated DGH showed a better solubility and gelling ability than its CSPI counterpart, which suggested that the combination of selective proteolysis and HIU could be a novel method for soy protein modification.

## Figures and Tables

**Table 1:** The relative contents of the individual subunits and small protein fractions in CSPI and DGH according to the densitometric analysis of SDS-PAGE.

Sample	CSPI		DGH	
Band	MW (kDa)	Content (%)	MW (kDa)	Content (%)
α’	79.0	9.3	80.2	13.2
α	70.4	13.6	71.5	25.7
β	49.5	10.6	50.4	20.1
A3	39.2	7.6	38.6	0.1
A1,2,4	33	27.6	32.5	1.9
Small fractions	32~22	4.2	32~22	1.2
B	19.7	25.5	20.1	0.5
Small fractions	<18.3	1.6	<18.3	37.3

**Table 2:** Effect of high-intensity ultrasound treatment (20 kHz at 400 W for 0, 5, 20, 40 min) on the average particle size (Z-average) and polydispersity index of CSPI and DGH.

Sample	CSPI		DGH	
	Z-Average (nm)	PDI	Z-Average (nm)	PDI
0 min	66.09 ± 4.93 ^a^	0.428 ± 0.040 ^b^	48.90 ± 0.45 ^a^	0.897 ± 0.108 ^b^
5 min	90.52 ± 1.13 ^c^	0.341 ± 0.033 ^a^	119.57 ± 1.36 ^c^	0.321 ± 0.038 ^a^
20 min	72.10 ± 0.03 ^b^	0.382 ± 0.012 ^ab^	131.90 ± 1.64 ^d^	0.308 ± 0.010 ^a^
40 min	67.42 ± 0.19 ^ab^	0.374 ± 0.012 ^a^	75.29 ± 1.59 ^b^	0.412 ± 0.028 ^a^

Values are means ± standard deviation. Different lowercase letters (a, b, c, d) indicate significant differences (*p* < 0.05, Duncan′s test) between high-intensity ultrasound HIU times in the same column.

**Table 3:** Effect of high-intensity ultrasound treatment (20 kHz at 400 W for 0, 5, 20, 40 min) on the secondary structure of CSPI and DGH.

**Composition (%)**	**CSPI-0 min**	**CSPI-5 min**	**CSPI-20 min**	**CSPI-40 min**
α-helix structure	0.83 ± 0.25 ^a^	8.18 ± 0.39 ^b^	9.10 ± 0.14 ^c^	9.03 ± 0.07 ^c^
β-sheet structure	66.43 ± 0.29 ^c^	61.20 ± 0.87 ^b^	57.35 ± 0.49 ^a^	59.60 ± 1.77 ^b^
β-turn structure	0.00 ± 0.00 ^a^	1.08 ± 0.38 ^ab^	3.35 ± 0.78 ^c^	1.80 ± 0.92 ^b^
Random coil structure	32.74 ± 0.53 ^b^	29.54 ± 0.28 ^a^	30.20 ± 0.14 ^a^	29.57 ± 0.78 ^a^
Ordered structure	67.26 ± 0.53 ^ab^	69.38 ± 0.65 ^c^	66.45 ± 0.64 ^a^	68.63 ± 1.70 ^bc^
Unordered structure	32.74 ± 0.53 ^bc^	30.62 ± 0.65 ^a^	33.55 ± 0.64 ^c^	31.37 ± 1.70 ^ab^
**Composition (%)**	**DGH-0 min**	**DGH-5 min**	**DGH-20 min**	**DGH-40 min**
α-helix structure	0.33 ± 0.21 ^a^	7.19 ± 0.24 ^b^	6.83 ± 0.23 ^b^	7.40 ± 0.44 ^b^
β-sheet structure	60.23 ± 0.57 ^a^	63.00 ± 0.68 ^b^	64.90 ± 0.72 ^c^	63.67 ± 0.87 ^bc^
β-turn structure	0.00 ± 0.00 ^a^	0.15 ± 0.12 ^ab^	0.23 ± 0.14 ^ab^	0.50 ± 0.46 ^b^
Random coil structure	39.44 ± 0.50 ^c^	29.66 ± 0.34 ^b^	28.04 ± 0.59 ^a^	28.43 ± 0.06 ^a^
Ordered structure	60.56 ± 0.50 ^a^	70.19 ± 0.44 ^b^	71.73 ± 0.58 ^c^	71.07 ± 0.50 ^bc^
Unordered structure	39.44 ± 0.50 ^c^	29.81 ± 0.44 ^b^	28.27 ± 0.58 ^a^	28.93 ± 0.50 ^ab^

Values are means ± standard deviation. Different lowercase letters (a, b, c, d) indicate significant differences (*p* < 0.05, Duncan′s test) between HIU times in the same row.

**Table 4:** Effect of high-intensity ultrasound treatment (20 kHz at 400 W for 0, 5, 20, 40 min) on the λ_max_ and fluorescence intensity (FI) of CSPI and DGH.

Sample	CSPI		DGH	
	λ_max_ (nm)	FI (a.u.)	λ_max_ (nm)	FI (a.u.)
0 min	337.4	5311.7 ± 10.6 ^b^	338.6	5085.3 ± 24.0 ^a^
5 min	335.0	5493.3 ± 46.7 ^c^	333.4	5668.7 ± 18.4 ^d^
20 min	333.6	7126.0 ± 31.1 ^g^	332.4	7244.0 ± 32.5 ^h^
40 min	333.6	6371.7 ± 53.0 ^f^	332.6	6040.7 ± 15.5 ^e^

Intensity values are means ± standard deviation. Different lowercase letters (a, b, c...) indicate significant differences (*p* < 0.05, Duncan′s test) between the fluorescence intensity of different samples.

**Table 5:** Normalized intensity values at selected regions of the FT-Raman spectra of high-intensity ultrasound (HIU; 20 kHz at 400 W for 0, 5, 20, 40 min) treated CSPI and DGH.

**Band Assignment ** **[Wavenumber (cm^−1^)]**	**CSPI-0 min**	**CSPI-5 min**	**CSPI-20 min**	**CSPI-40 min**
S-S stretching (g-g-g) [500–510]	1.02 ± 0.02 ^b^	0.97 ± 0.01 ^a^	0.97 ± 0.02 ^a^	0.95 ± 0.03 ^a^
S-S stretching (g-g-t) [515–530]	0.93 ± 0.02 ^a^	0.99 ± 0.02 ^b^	1.01 ± 0.03 ^b^	0.99 ± 0.01 ^b^
S-S stretching (t-g-t) [535–545]	0.91 ± 0.01 ^a^	0.97 ± 0.01 ^b^	0.96 ± 0.02 ^b^	0.97 ± 0.03 ^b^
Tyrosine doublet [850 cm−1/830]	1.07 ± 0.02 ^d^	0.93 ± 0.02 ^a^	0.97 ± 0.01 ^b^	1.01 ± 0.03 ^c^
C-H2 bending [1448–1452]	1.11 ± 0.03 ^b^	1.03 ± 0.02 ^a^	1.09 ± 0.03 ^b^	1.13 ± 0.04 ^b^
C-H stretching [2929–2937]	1.70 ± 0.04 ^b^	1.55 ± 0.05 ^a^	1.69 ± 0.04 ^b^	1.68 ± 0.03 ^b^
**Band Assignment ** **[Wavenumber (cm^−1^)]**	**DGH-0 min**	**DGH-5 min**	**DGH-20 min**	**DGH-40 min**
S-S stretching (g-g-g) [500–510]	1.03 ± 0.04 ^c^	0.94 ± 0.01 ^ab^	0.96 ± 0.02 ^b^	0.90 ± 0.03 ^a^
S-S stretching (g-g-t) [515–530]	0.91 ± 0.02 ^a^	1.05 ± 0.02 ^b^	1.13 ± 0.05 ^c^	0.99 ± 0.04 ^b^
S-S stretching (t-g-t) [535–545]	0.88 ± 0.01 ^a^	1.04 ± 0.03 ^c^	1.11 ± 0.04 ^d^	0.96 ± 0.04 ^b^
Tyrosine doublet [850 cm−1/830]	1.14 ± 0.04 ^c^	0.96 ± 0.02 ^b^	0.88 ± 0.02 ^a^	1.03 ± 0.06 ^b^
C-H2 bending [1448–1452]	1.23 ± 0.05 ^c^	1.16 ± 0.03 ^b^	1.01 ± 0.02 ^a^	1.17 ± 0.02 ^bc^
C-H stretching [2929–2937]	1.84 ± 0.08 ^c^	1.62 ± 0.05 ^b^	1.44 ± 0.03 ^a^	1.70 ± 0.06 ^b^

Values are means ± standard deviation. Different lowercase letters (a, b, c, d) indicate significant differences (*p* < 0.05, Duncan′s test) between the HIU times. g-g-g is short for gauche-gauche-gauche, g-g-t is short for gauche-gauche-trans, t-g-t is short for trans-gauche-trans.
